# Report of Committee on Medical Sciences

**Published:** 1851-01

**Authors:** 


### ARTICLE I.

*Notice of the Report of the Committee on Medical Sciences,* of which Usher Parsons, M. D., of Rhode Island, was Chairman. By A. B. Palmer, M. D., of Chicago, Ill.

This report, occupying fifty pages of the published transactions of the National Association, for 1850, is before us, and we are happy to find that the Committee not following the course of their predecessors, have obeyed the "regulations" under which they were appointed, and have confined themselves to the consideration of "the progress of the Medical Sciences in *America* during the year of their service."

By treating only of subjects assigned to them, and confining their researches to this country, and the year of their service, their report is rendered much shorter than that of the preceding year—a circumstance we do not regret. These reports have been too lengthy to be listened to by the Association before being referred to the Committee on Publication, thereby endangering the putting forth of sentiments not approved by that body; and they have been too voluminous to be embraced within a volume of convenient size. We have brought to us in the various foreign Retrospects and Abstracts, a very perfect synopsis of the improvements in the various departments of the profession abroad, rendering their re-publication here unnecessary; while the accounts of the improvements in our own country are scattered through numerous local journals, or exist only in the private note-books of experimenters and observers, and require to be judiciously collated, for preservation and reference. We agree with the committee in the opinion, that there is a greater evil than the inconvenient length of the reports, in the introduction of maters from abroad. "It impairs the vigilance and industry necessary to the accumulation of intelligence purely American." They justly say, "in a country so widely extended, it is a laborious task to gather materials for such a purpose. Many interesting facts must occur which never come to the knowledge of a committe, even when foreign matter is excluded, and still more must this be the case when the labor of collecting domestic matter can be diminished by substituting foreign."

We cannot but express the hope that the example of this committee in strictly adhering to the letter and spirit of the law of the Association will be followed by all the committees of that body hereafter.

In pursuing the proper subject of the report, the committee have contented themselves with presenting an abstract of the labors and conclusions of others without often making suggestions or expressing opinions of their own.

Under the head of *Anatomy* and *Physiology*, the first subject referred to, is the "Arrangement of the Cancelli in Bones," by that very excellent Anatomist, Prof. Jeffries Wyman of Harvard University. His general conclusions are thus briefly stated:

"1. The cancelli of such bones as assist in supporting the weight of the body, are arranged either in the direction of this weight, or in such a manner as to support and brace those cancelli which are in that direction. In a mechanical point of view, they may be regarded in nearly all these bone as a series of 'studs' and 'braces.'

"2. The direction of these fibres in some of the bones of the human skeleton is characteristic, and it is believed has a definite relation to the erect position, which is naturally assumed by man alone."

It would âppear from the statements and illustrations given, that in the minute structure of the bones, as indeed is the case in every part of nature, the most accurate adaptation of means to ends is observed. To this conclusion, as we advance in the knoweldge of nature, we should expect to be brought ever; yet each new illustration of the principle of universal adaptation and design, adds an item to its confirmation.

The next subject presented in the report, is the "Capacity of Crania in the different races of Man," as shown by the investigations of Dr. Samuel G. Morton, Author of "*Crania Americana*:" but as these facts have been so generally published in the Journals, specifications of them here are not deemed necessary.

While the researches of Dr. Morton have shown that the capacity of the Negro Cranium is greater than that of several other varieties of the "family of man," some observations of Dr. John Neill of Philadelphia, next referred to in the report, seem to indicate that the African race is a lower variety of the species than any of the others whose skulls are found in the immense collection of Dr. Morton which adorn the Halls of the American Academy of Natural Sciences. The principle and facts from which this conclusion is drawn, are the following:

Professor Agassiz has propounded the law, that "in the different formations through which animals pass, from the first formation in the embryo up to the full grown condition, may be found a natural scale, by which to measure and estimate the position to ascribe to any animals." According to this law, the persistence of a fœtal stage of formation in the adult, would mark the race to which it was peculiar as a lower variety. Dr. Neill has shown by extended observation, that certain embroyic conditions continue in the African cranium with vastly greater frequency than in any other race which has been examined; and that these conditions are not found at all in the crania of Caucasians.

One of these fœtal peculiarities, is "the division of the articulating surfarce of the occipital condyle into two faces by a transverse ridge or groove." Another is, "the absence of a sharp edge at the lower boundary of the anterior nares, running from the superior edge of the nasal process to the anterior nasal spine," and other bones of the Negro skull, Dr. N. alledges, exhibit similar facts.

Of the correctness and and universal applicability of the law stated by Prof. Agassiz, and of the significance and importance of these alledged peculiarities in the bones of the African head, we are not prepared to express an opinion, but will venture the remark that the position which the Negro should occupy in the scale of rational and social beings, must be determined by other circumstances than such slight anotomical peculiarties as are designated by Dr. Neill. These, with other physical peculiarities, however, will have their influence in determining his position in natural history.

We further learn on the subject of the different Races of Man, from our report, that P. A. Brown, L. L. D., of Philadelphia, maintains in oppostion to the opinion of Dr. Prichard, that the covering of the head of the Negro is wool and not hair. Dr. B. sustains his views by microscopic observations according to which, hair is more complex in its structure than wool.

An improved method of vascular injections for Microscopic Researches, by Dr. P. B. Goddard of Phila., and a new mode of "Direct Exploration of the Eye," by Dr. T. Wood of Cincinnati, are briefly referred to.

Dr. Bennett Dowler's experiments of the Motor Powers, made by vivisections of the "Great Saurian of Louisiana," and the singular results produced, are next noticed. With these, the readers of this Journal have been already furnished. The committee say, that "Dr. Dowler is a bold experimenter, and his laudable zeal and industry have already added many valuable facts to medical science, and we doubt not his future labors will either strengthen or correct the opinions he has advanced." As the task of drawing up the next Report on Medical Science will devolve on Dr. Dowler, we shall expect that his labors in his favorite field, during the present year, will not be abated.

The report states that on the subject of the Formation and Developement of Organic Cells, the essential organic element of animal and vegetable bodies, some of the statements of Schwann and Schleiden, which have been the received doctrines since their discoveries, as applicable to all living forms, has been questioned by Dr. W. J. Burnett, in a communication laid before the American Association for the Advancement of Science, at its late meeting in Cambridge.

The statement of the German discoverers is, that nuclei ap-appear in organisable liquid, and that the growth of cells is *upon* and *around* these nuclei, while Dr. Burnett's observations on epithelial cells of the mucous membranes, and on pus-cells, the result of inflamation, have brought him to the opinion, that the nuclei themselves expand into hollow spheres, containing a fluid at first clear, then cloudy, at length giving rise to nuclei, developing in this way a neucleated cell. These nuclei expand into cells as before, bursting the original one, becoming themselves possessed of nuclei, and thus the multiplcation and growth goes on. These discrepancies of opinions among microscopic observers, upon points hitherto considered sufficiently established, must tend to destroy confidence in the precision and accuracy of their observations.

We next find a notice of some observations by Prof. J. W. Draper of N. Y., on "Allotropism in the Living Organic Elements," which we regard of sufficient interest to introduce entire.

"It is not necessary to explain the meaning of this term, which is applied to ignorganic and elementary bodies that appear in differen forms, assuming different physicsl and chemical relations. The three leading neutral nitrogenized bodies, fibrin, albumen and casein, are in a remarkable manner allotropic. First, they are mutually convertible, hardly deserving to be called distinct substances more than charcoal, plumbago, and diamond, allotropic forms of carbon. Again, each one of them easily assumes new forms; one variety of fibrin being soluble in water, another being not so, &c.

"Of the agents which, in the inorganic kingdom bring about these changes, the imponderable principles are the most important. Dr. Draper believes that the nervous system has a similar power of changing the allotropic condition of these proximate elements of the body, and that the existing allotropic state of an organized molecule determines whether it shall yield to the oxidizing influence of arterial blood, or not, 'that this is the fundamental fact on which all the laws of intertistial death depend.' He suggest that inflamation may begin by an allotropic change in the soft solids of the part, disposing to more active oxidation. Hence, according to the laws of circulation he has formerly so beautifully developed, an increased afflux of blood would result. Hence, too, a higher proportion in the urine of urea and sulphuric acid, results of the oxidation of the proximate elements above named. In congestion, these conditions are all reversed. This view seems to harmonize with the facts, and presents them more clearly to our minds."

The report proceeds to state some experiments of Prof. J. Blake of St. Louis, for the purpose of ascertaining the quantity of blood in the body, which led the experimenter to conclude that the quantity is less than has been supposed. The weight of the blood of a Dog, the animal experimented upon, is estimated at one-eigth, or one-ninth of that of the whole animal.

The subject or Lymph-Corpuscles being visible in Dried Blood under the Microscope after moisteing, and where the colored blood-discs could not be recognized; of the relation of the Nitrogen of the Air to Respiration and Voice, by Prof. Moultrie, and of Parasitic Life by Dr. Leidy of Philadelphia, and Dr. Bowditch of Boston, are successively referred to. A practical remark respecting the animal and vegetable parasites that affect the human teeth, by Dr. Bowditch, is worrthy of record—it is that Tobacco Juice and Smoke did not impair their vitality, neither did most of the popular toothwashes, while *Soap* destroyed them instantly. Pure White Soap he therefore recommendsa s by far the best substance for cleansing the teeth.

The subject of Endemic, Epidemic and Contagious Diseases, next occupies the attention of the committee. The work of Prof. J. K. Mitchell on the cryptogamic origin of Malarial and Epidemic Diseases is named, and the pamphlet of Prof. J. Knight, on the propagation of communicable Diseases, and the articles of Prof. S. H. Dickson on the same subject are epitomised. The conclusions to which Dr. Knight arrives on this subject, are briefly stated as follows.

"1st. All febrile diseases, of a typhoid and malignant type, depend upon, as their predisposing cause, a certain endemic or epidemic constitution of the atmosphere. What this state is, is entirely unknown.

"2d. This epidemic constitution, aided by the common exciting causes of disease, is at times sufficient to produce them.

"3d. Whenever this epidemic constitution is present, they will be communicated from the sick to the well, and the communicability is a common cause of their propagation."

Prof. Dickson mentions two essential characters of contagion. "The matter which produces such disease, must be germinal and self-multiplying, and this reproduction must depend upon or be favored by the very process of disease which itself gives rise to. The essential matter of contagion is not isolated and examined by itself, and is too minute to be studied with the microscope. It exists in the cells of cancers—in the newly produced fluids of the exanthemati—in the pus-globules and ichor of certain specific diseases—in conection with animalcula or fungus vegetation—and lastly, diffused through the blood or certain normal secretions. But it is distinct from all these visible forms which accompany it—it is contained in these fluids—associated with these organic forms, but is not identical with them. In febrile contagious diseases the contagious matter radiates, but in non-febrile contagious diseases, contact is necessary to propogation. He further suggests that *perhaps* the germ of variola may grow and multiply in another nidus than the human body, which is its [illegible] habitation."

With this view of contagion, he regards typhus, pertussis, puerperal fever, dysentery, yellow fever and cholera, as they become epidemic, are self-propagating, and easily spreading from a focus, as necessarily contagious. He particularly insists upon this being the case in regard to cholera, and that its germ is proved to be organic and have its nidus in the human body. In the case of typhus, pertussis, puerperal fever and dysentery, and we might add erysipelas, facts decidedly favor this view; but in the case of cholera, the writer of this article is decidedly of another opinion.

On the subject of contagion and infection, there seems much vagueness of thought and expression with many writers, and we are sorry to class Dr. Knight among the number. Dr. Dickson goes farther down to the foundation of his subject, and considers the question of the cause and essential nature of contagion, and properly, as we think, places it in a material and specific poison—a substance, the particles of which are too minute to be seen by glasses, but which nevertheless have a distinct physical existence, probably organic. We place the cause of infectious diseases in a similar *Materia Morbi*; but we, unlike Dr. Dickson, make a distinction between infection and strict contagion; and our distinction is this—that in a strictly contagious disease the *Materies Morbi* is ordinarily produced and multiplied only in the bodies of persons laboring under the disease, while in an *infectious* disease the material cause is produced and multiplied exterior to, and independent of the body in a state of disease. In this latter class we place the poison which produces Cholera, and we therefore consider this an infectious, not a contagious disease. The poison of an infectious disease may be portable—may be carried on ship-board from New York to Folly Island, or New Orleans, as it has been carried—or may be spread by its own independent and inherent powers of multiplication, when circumstances are favorable, for many miles across uninhabitated and untraveled deserts, as we believe it has been known to have been. It may select particular localities in a city—particular sides of streets—particular stories of buildings—like swarms of insects or clouds of fungous sporules—may appear most abundant where conditions of soil, atmosphere, moisture, electricity, &c., are most favorable for its existence and multiplication—it may spread rapidly and exist in large quantities where these are favorable—or if germs be brought into localities in these respects unfavorable, they may fail to multiply, or succeed to so limited an extent as to show only slight signs of their existence, affecting only persons most strongly predisposed, and soon disappear. We shall not attempt to say what this poison is—whether an *animacule*—a vegetable sporule—an organized germ cell, or a chemical product formed from elements in the atmosphere. One of the four we conceive it must be, which, we do not know; but of one thing we are confident, that upon the hypothesis of a material poison, as the cause of cholera, produced and multiplied independently of the bodies of the sick, *all* the facts in this widely spread, terribly fatal, and ever identical disease, can be accounted for; and *only* upon this hypothesis *can* they be accounted for.

As a single argument against the doctrine of the contagion of cholera, we mention the fact sufficiently attested by observation, that in a locality where the disease is prevailing, those in close proximity and contact with the sick, are not a whit more liable to an attack, than those who avoid such proximity and contact. Location makes a difference, but the presence of the sick does not. There is not, to our mind, the slightest evidence, that, as in the case of small-pox, the poison is produced in the bodies of the sick, and communicated from their bodies to others, which is the essential quality of contaion, but all the evidence tends to the conclusion that it is not.

These remarks may be considered dogmatical; assertions without proof; but we must be content for the present with a mere statement of some of our opinions on this subject, not having space in this article for an extended reference to facts, or for a course of arguments to illucidate and sustain them.

Irregular Malarial Diseases by Prof. Kirtland; Re-Vaccination by the late Dr. Fisher of Boston; the Pathology of Cholera, from theexcellent "Report on the Cholera in Boston in 1849"; and from the committee appointed to examine the condition of the intestines in this disease, by the College of Physicians of Philadelphia, are next noticed by our committee; and although possessing interest, yet not much novelty, we are compelled for want of space to pass them over.

A valuable contribution from Prof. Austin Flint, on "Serous Effusions into the Cavity of the Arachnoid," is next presented. This pathological condition, Dr. Flint considers as having been generally and improperly overlooked of late; believing it, as he does, a frequent cause of death. The fluid during life gravitates towards the spine and presses on the Medulla Oblongata, producing derangement of the functions whose nerves are derived therefrom, viz: respiration and deglution. Dr. F. thinks this effusion liable to occur as an incident in the course of various affections, being immediately produced by celebral congestion, and without premonitory symptoms by which it can be predicted; that it is not an unfrequent cause of sudden deaths, such as are ascribed to cerebral congestion, or effusion within the ventricles; and that it causes death by *apnœa*, from pressure on the medulla oblongata.

It is distinguished by the sudden developement of grave cerebral symptoms as coma without paralysis; disordered respiration, without proportionate disturbance of circulation; lost or impaired deglutition without disorder of intellect, if the patient can be aroused—all these symptoms not preceded by signs of meningitis, and rapidly and almost surely tending to a fatal result by suspension of respiration. He expresses doubts whether much can be expected from treatment.

We regard the subject as one of much interest, and which requires further investigation.

A new form of Cerebral Disease, described by Dr. L. V. Bell of the McLean Hospital for the Insane, Massachusetts, is next presented; and as Dr. Bells observations are of much interest, and are well condensed by the committee, we shall give their language on the subject nearly entire. We the more readily give place to this, as those having charge of institutions for the Insane are constantly complaining of the mistakes of physicians in cases of insanity generally, and particularly in those of this kind. They state that cases are often sent to Hospitals which from their rapidity had better be kept at home, while others are not sent sufficiently soon; and they also complain of the too general, profuse and indiscriminate abstraction of blood, and the too free use of other depleting measures. Whether these complaints are in all cases, well founded, we are not prepared to say; but the fact that they are so generally made by those who make insanity a speciality, is sufficient to arrest the attention, and call for the deliberation of every medical man. The report says,

"This new form of disease, often witnessed in hospitals for the insane, which, though generally passing for acute mania, really differs from that and every other affection of the brain, and may be easily recognized after being once noticed. It is marked by the following peculiarities: It attacks abruptly, and generally within a week compels a resort to the hospital. The want of sleep has then become extremely urgent. There are no signs of undue arterial action in the pulse. The delirium is suspended for a moment or two by an effort of attention. There is a loss of appetite and loathing of food, accompanied with the idea of its being poisoned or filthy. The hallucinations are characterised by an indefinite sensation of distress and horror, and the patient is not easily soothed by management, as in other cerebral affections. Although resembling at first sight the advanced stages of typhoid fever, especially in its delirium, it has none of the characteristic external marks of this disease. It is for the most part fatal, and runs its course, whether to death or recovery, in three or four weeks. It differs from mania in the following particulars. The mental disturbance is rather the delirium of bodily disease than of mania. The loss of appetite, want of sleep, and especially the sudden loss of strength, are seldom witnessed together in mania. The rapidity of its course, too, may be considered as a diagnostic symptom. It is distinguished from delirium tremens by the absence of the cause; by the character of the hallucinations, which are indefinite impressions of personal danger and sinfulness, rather than serpents, devils, sheriffs, vermin, &c.; by the absence of the pathognomonic perspiration of delirium tremens; and by the fact that the latter is limited to eight or ten days, generally terminating in recovery after a long and refreshing slumber. From meningeal inflammation it differs, in wanting the pulse of inflamation, in the absence of pain in the head, of intolerance of light, and of high raving delirium, all which are usually present in meningitis. The only treatment which seems to promise relief, consists in opium, and the alcoholic and diffusible stimuli liberally used.*

The committee next refer to "Etherization in Insanity." It appears from the statements of Dr. Ray of Butler Hospital, R. I., corroborated by Dr. Bell, that only slightly beneficial effects are produced by it.

The report next gives a summary of a communication from Mr. J. B. Richards, Principal of the School for Teaching and Training of Idiots, at South Boston, Mass. This State usually among the first in the prosecution of every good work of Science and Benevolence, has established this experimental School, numbering last March, when we had the pleasure of visiting it, some twelve or fifteen children and young persons, selected with with a view to variety, from the different classes of idiots; ranging from those that are merely simpletons, down the lowest grade of helpless deformity, and pitiable amensia. The plan of management appears simple and philosophical; and the improvement made in the condition of many of those unfortunate beings, according to the statements of the Principal, who is evidently entirely devoted to his pursuit, is highly gratifying.

According to the communication of Mr. Richards to the

*American Journal of Insanity, October, 1849.

Committee, out of 403 cases of idocy investigated under the auspices of the State, 302 were congenital, throwing the responsibility of three fourths of the cases upon the parents, and the intimation is made that the condition of the remainder would have been greatly modified, had their parents possessed good constitutions, and observed the laws of health, as *every parent* should do, who takes the responsibility of bringing a human being into existence.

The report proceeds: "Believing that idocy results in a great measure from a low condition of the parents' vital forces, Mr. Richards enumerates the following agents which produce this condition; alcoholic drinks, licentiousness in all its forms, gluttony, exhaustion from too great mental or physical labor, weakness resulting from the want of sufficient mental or bodily exercise, low and insufficient diet, and unhealthy occupations."

A tabular view of a large number of cases showing their particular causes so far as could be ascertained is given—but our space will not allow us to transcribe it. In those cases not congenital, perverted sexuality is by far the most frequent cause.

We have traveled somewhat out of our way in noticing this school for the education of idiots, because these unfortunate beings, many of them susceptible of great improvement, have been almost universally neglected, so far as public systematic efforts are concerned, to improve their condition, this institution being the first of the kind, at least of a public character, ever undertaken in this country.

*Neo-macropia*—or a peculiar kind of abnormal vision not before noticed, by Prof. C. Dewey; a case of *Spontaneous Hydrophobia*, by Dr. Condie; Augina Pectoris. by Dr. S. Kneeland of Boston; Cerebral Symptoms in Heart Disease, by Drs. Flint, and D. J. C. Cain, are next successively noticed. On the subject of the pathology of Augina Pectaris, Dr. Kneeland comes to the conclusion that the disease consists in derangement of the *moter filements* of the par vagum, and that this nerve may be affected with "Neuralgia and rheumatism, with inflamation, it may be compressed by morbid growths; its spinal origin may be compromised by hemorrhage, accidental wounds, and various irritations, all of which may cause the symptoms of this affection." He adds that "in addition to the remedies of the books, special attention should be given to the inhalation of oxygen, and to the use of electricity."

The causes of Pulmonary Consumption are next briefly referred to. Dr. Condie stated, that "since more attention had been paid to the collection of correct statistics, consumption has been shown to be the disease rather of civilized life than of locality or climate." High southern, and high northern latitudes are now considered nearly alike favorable to the developement of the disease. "Besides hereditary influence, long-continued sedentary occupations, indoor confinement, particularly in narrow, ill-ventilated apartments; insufficient nutriment, close mental occupation, unbroken by relaxation and active exercise, and anxiety of mind," are reckoned among the most prominent causes of the disease. These latter views, though not novel, cannot be too frequently reiterated, or too constantly borne in mind. The great relative importance of hygienic management in the treatment, as well as prevention of this disease—is now universally acknowledged.

Prof. C. Hooker's essay on Intestinal Auscultation, favorably noticed by the committee, we must pass over, as well as Dr. Breed's experiments with plasters of cantharides, hot water and steam, combined with pressure, upon his own flesh.

We will simply state, however, that these experiments brought Dr. Breed to the following conclusions: "1st. That evaporation is not essential to the formation of a blister."

"2d. That a certain degree of pressure will prevent the formation of a blister." And they brought us to this conclusion, that Dr. Breed of Cambridge Laboratory, is *warmly* engaged in the cause of science.

We can only name several other subjects of more or less interest, treated of in the report, viz: "Urinary Calculi"—"Causes of Mortality in Children of the two sexes;" "Influences operating to change the number of births;" "Mode of Actions of Poisons;" under which last head the continued experiments of Dr. Blake of St. Louis, calculated to show that poisons act alone by absorption, are mentioned. "Poisoning by Cedar Oil;" "Effects of Strychinia and Hydrocyanic Acid;" "Chloroform as an antidote to Strychinia;" under which head it is stated that two drachms of Chloroform swallowed, counteracted in fifteen minutes the manifested effects of three grains of Strychnia in Solution. "Comparative effects of different Anæsthetics;" "effects of Anæsthetics on Vegetable Organs;" "Action of different waters on Lead Pipes;" "Relation of Lead to Air and Water;" and "Moisture Ammonia, and Organic matter in the Atmosphere."

The last subject of science dwelt upon in the report, is the much talked of Ozone. On account of the interest this subject may naturally be supposed to possess with the readers of this Journal, we give the language of the committee upon it entire.

"This substance has come into notice during the year past from its supposed connection with catarrhal diseases, as noticed in Europe; and with cholera, as suggested by physicians of Chicago. The nature and even the individuality of ozone are not quite determined. Professor Peter has published interesting observations on this subject. He tested the amount of ozone in the air at Lexington by the usual means, a strip of bibulous paper being smeared with a paste of starch and a solution of pure iodide of potassium. His observations were daily, from June 30 to August 12, being not far from the complete period in which cholera prevailed at Lexington. There was no constant relation between the probable amount of ozone in the air and the daily number of deaths; the indications of ozone did not cease with the cessation of cholera; and it would seem that the principle which discolors the iodide of potassium is rarely absent from the atmosphere.

"Some considerations unfavorable to the supposed influence of ozone are these: Ozone is a vaporous or gaseous substance and must tend to diffuse itself equally, which the cholera does not. If ozone is produced by electrical causes, it certainly must be present in the atmosphere of South America, which is known to be remarkably exempt from the epidemic. As the slow combustion of phosphoros produces ozone, workmen in manufactories of friction matches should suffer from the diseases produced by it. The ozone theory of cholera, on the whole, is not sustained.*

Professor Ellet made analyses of the air in New York, under the auspices of the Board of Health, during the prevalence of cholera in that city. His results were wholly negative in regard to the prevalence of any foreign matter in the air. As to ozone, he says, 'I was forced to the conclusion not only that no such peculiar principle or condition existed in the atmosphere at the time, but that the experiments of those European chemists who have announced the production, by artificial means, of such a new form of matter, or such a modified or 'allotropic' condition of any of those forms previously known to us, are unsatisfactory.'†

Prof. Horsford has observed that ozone, subjected to a heat of 130° Fahrenheit, loses its properties.

The report closes by recommending the restriction of the future committees of the Association, to the letter of its existing regulations, viz: *to improvements in the respective branches assigned them, during the year of their service*; (with which sentiment we are constrained to repeat our hearty concurrence;) and by an allusion to the increasing extent of our country, and its multiplying facilities for scientific advancement. Agassiz, Guyot, Horsford, Wyman, and Leidy, are alluded to, (and there might have been added Clark, and Draper, and many others,) as those whose light like the rising sun, is gilding the east, and spreading its beams over our wide-spread country. Considering the impression which many otherwise intelligent

*Transylvania Medical Journal, Oct. 1849, p. 148.

†Report of Sanitary Committee on Cholera in New York, 1849, page 59.

people of the Atlantic border seem to entertain of the west, the following recognition of its *advancing civilization*, coming, as it is presumed, from the chairman of the committee, shonld pass unnoticed.

"Already," continues the report, "in the Valley of the Mississippi, where within the memory of the living, the red man was lord of the domain ; beautiful cities adorn the banks ot the majestic rivers," (and there might have been added, populous ports grace the inland seas,) "containing Colleges, and Seminaries of learning, where gifted minds are diffusing the blessings of science and literature."

While our brethren of the east claim to compare themselves with the rising *Sun*; we hope that the mighty west, with her Drake, her Dudley, her Dowler, and her host of others, may not be considered as presumptuous, in claiming some *Stars* in the firmament of American Medical Science.

On the whole we are pleased with the document, the substance of a large portion of which, we have attempted to present. A greater amount of originality of thought, and of critcal remarks, indicating the judgment of the committee, as to the truthfulness and relative importance of the various alleged discoveries and improvements, would have been desirable, giving the whole less the appearance of a mere abstract, and more the character of an elaborate philosophical report emanating from a learned society. But the American association is in its infancy, and we can hardly expect as much from it at present, as from the older European Societies of a similar character. It will improve in the tone and general character of its eminations as it advances. In the present instance, a laudable amount of industry has been manifested by the committee in collecting materials, and the style of their report for the most part, is concise, perspicuous, and correct.

